# Can cyclone exposure explain behavioural and demographic variation among lemur species?

**DOI:** 10.1371/journal.pone.0300972

**Published:** 2024-03-27

**Authors:** Alison M. Behie, Travis S. Steffens, Keaghan Yaxley, Alan Vincent, Patricia C. Wright, Steig E. Johnson, Mary S. M. Pavelka

**Affiliations:** 1 School of Archaeology and Anthropology, Australian National University, Canberra, Australian Capital Territory, Australia; 2 Department of Sociology and Anthropology, University of Guelph, MacKinnon, Guelph, Canada; 3 Planet Madagascar, Guelph, Ontario, Canada; 4 Research School of Biological Sciences, Australian National University, Canberra, Australian Capital Territory, Australia; 5 Division of Evolution, Ecology & Genetics, Research School of Biology, Australian National University, Canberra, Australian Capital Territory, Australia; 6 Environmental Education Department, Centre ValBio, Ranomafana, Ifanadiana, Madagascar; 7 Department of Anthropology, Stony Brook University, Stony Brook, New York, United States of America; 8 Department of Anthropology and Archaeology, University of Calgary, Calgary, Alberta, Canada; Universidad de Guadalajara, MEXICO

## Abstract

Madagascar has a harsh and stochastic climate because of regular natural disturbances. This history of regular cyclones has been hypothesised to have directed evolutionary changes to lemur behaviour and morphology that make them more resilient to sudden environmental change. These adaptations may include: small group sizes, high degrees of energy-conserving behaviours, generalist habitat use, small home ranges, small body size, and a limited number of frugivorous species. To date, however, no one has tested how variation in cyclone exposure across Madagascar is associated with variation in these resilience traits. In this study, we created a detailed cyclone impact map for Madagascar using Koppen-Geiger climate class, historical cyclone tracks, the Saffir Class of cyclone and hurricane intensity, and precipitation data. We also used existing literature to calculate a resilience score for 26 lemur species for which data existed on resilience traits. Our cyclone impact map was then overlaid on known geographic ranges of these species and compared to resilience score while controlling for phylogenetic non-independence and spatial autocorrelation. We found no association between cyclone impact in a lemur range and their resilience score. When assessing traits individually, however, we found that cyclone impact was positively associated with body size, suggesting that the more impacted a species is by cyclones the smaller they are. We also found cyclone impact to be negatively associated with frugivory, with species in higher impact zones eating more fruit. While unexpected, this could reflect an increased production in fruit in tree fall gaps following cyclones. While we did not find a pattern between cyclone impact and behavioural resilience in lemurs, we suggest a similar study at a global scale across all primates would allow for more taxonomic variation and reveal larger patterns key to understanding past and future vulnerability to natural disturbances in primates.

## Introduction

Tropical storm activity has been on the rise since the mid-1970s, and recent modelling predicts still further increases in intensity and frequency, alongside an increase in global mean temperatures [1, 2]. Most non-human primate (hereafter primate) species live in the tropics and, if near the coast, are at risk of impacts from tropical storms [3]. The island of Madagascar, located in the South-West Indian Ocean cyclone basin is the primate habitat most impacted by such storms, being hit by 69 cyclones from 1912–2022 [4]. Also threatened with high rates of deforestation, Madagascar’s endemic lemur species have become one of the most endangered group of mammals globally [5]. However, despite frequent cyclone exposure, most of the research on what is driving lemurs towards extinction has focused on the current and historical impact of anthropogenic threats [5–7], with few studies exploring how cyclones have historically impacted the behaviour, biology and ecology of lemur species [8–10]. Nonetheless, it has been hypothesised that one of the main reasons why lemurs show differences in many traits compared to other primates is the unpredictable and harsh environment created in part by frequent cyclones [11].

Such an idea has been supported in Central American monkeys, where black howler monkeys (*Alouatta pigra*), exposed to significantly more hurricanes per kilometre of coastline within their overall range, live in significantly smaller groups and show more energy-conserving behaviours than the closely related mantled howlers (*A*. *palliata*, which are exposed to fewer hurricanes) [12]. As severe storms often cause prolonged and severe reductions in primate food supplies [13], reducing group size is a potential mechanism to cope with sporadically changing food supplies. Thus, severe weather events may be placing upper limits on how many animals could successfully live and breed in a group at one time. Similarly, an increase in time spent inactive and a reduction in time spent feeding, such as that seen in black howler monkeys following translocation to a new environment [14] or exposure to a hurricane [15], would allow for animals to better conserve energy and survive these periods of low food availability. It is also thought that, in lemurs, this capacity has extended to include torpor and hibernation as well as lower basal metabolic rates compared to other similarly sized primates [16].

Other variables thought to offer resilience following extreme habitat change include energy conserving behaviours, relying less on fruit, being a habitat generalist and having a small home range [11]. As fruit is often not produced for months or years following an extreme habitat change event (e.g., hurricane; [17]), specialist species that are constrained to specific dietary requirements may become more vulnerable to nutritional stress [13, 18–20] following a period of low fruit availability. Thus, relying less on fruit over the long term would allow for less disruptions to normal diets in stochastic environments. Dietary specialisation was associated with hurricane-driven population decline in terrestrial mammals including primates in Mexico [21]. Habitat specialists were more vulnerable to hurricanes as these species are less able to adjust to a different habitat type when their preferred habitat is damaged [21]. Arboreal primates are at higher risk because they are restricted to trees and would be more impacted when canopies are destroyed through hurricane damage [16]. Smaller home range size has been associated with better resilience to habitat change as species with large home ranges likely also have more ecological requirements, which will become harder to meet after a severe habitat disruption like a severe windstorm [22].

The history of regular cyclones in Madagascar is suspected to have resulted in changes to lemur behaviour and morphology, including small group size, high degrees of energy-conserving behaviours (including torpor), and a limited number of species that are dedicated frugivores [11]. Despite this, no study has yet to compare variation in these traits among lemur species against variation in cyclone impact across the island to determine if lemur species whose ranges are historically exposed to more cyclones show a higher incidence of predicted traits. In this study, we compare variation in six traits associated with cyclone exposure: group size, fruit consumption, energy conserving behaviours, territoriality, terrestriality, body weight and home range size with variation in cyclone exposure across Madagascar. We assess this relationship through a comparison of individual traits as well as the creation of a resilience score whereby traits are combined numerically to determine which species have the most traits expected to be resilient to cyclone exposure. We predict that species with higher resilience scores will be found in areas of higher historical cyclone exposure than species with lower resilience scores. Results of this study can be used to better understand how long-term exposure to cyclones across Madagascar may have impacted evolution in lemur species. Additionally, given expected increase with global climate change, this will help predict and mitigate future impacts on an already vulnerable group of animals.

## Methods

### Cyclone impact index map

We modelled cyclone impact using tropical storm data sourced from the National Oceanic and Atmospheric Administration (NoAA). This dataset (IBTrACS v04, 2021, [23, 24]) consisted of a worldwide spatial dataset of tropical storm tracks and their associated Saffir-Simpson class of storm intensity (0 represents tropical storm and 1–5 represents cyclone category). The data coverage for the study area (Madagascar) included tropical storm and cyclone tracks (n = 3548) that occurred between 1963 and 2021. Because tropical storms are classed as a zero in the dataset and cyclones ranked between 1–5, we transformed the Saffir-Simpson class data using the calculate function (n+1) in ArcGIS Pro. All track data beyond approximately 500 km of the island and track data not consisting of a tropical storm (wind speed less than 34 knots) were excluded. This procedure generated a local representation of all cyclone and tropical storms recorded around the island.

To model cyclone impact on Madagascar, a grid layer of 50 x 50 km polygon squares was projected over the island of Madagascar. A buffered spatial join operation was applied to the grid layer using the cyclone track layer as the input. For this operation we ran the spatial join operation multiple times to generate a range of summary statistics (sum, mean, mode, median, range, standard deviation, count and minimum and maximum cyclone impact) suitable for further analysis. Because there is some uncertainty in the impact radius of cyclones and storm size can vary significantly [25], we calculated the scale of Cyclone Impact score (for each summary statistic above) using three different impact diameters for each grid square, 87.5km, 175km and 350km (impact^87.5km^, impact^175km^, impact^350km^). These extracted values (Cyclone Impact) reflect an index of the overall impact of historical cyclones on each grid square by combining both intensity and frequency of historical cyclones.

To determine the Cyclone Impact on 133 lemur population ranges, a spatial join operation was applied to each species range using the Cyclone Impact grid layer as input (from above). Lemur species ranges were defined by a feature layer consisting of polygons representing the geographic range of each species sourced from the IUCN Redlist website as a shape file (accessed 4 August 2021). The above summary statistics consisted of aggregated values from each intersecting grid square of the Cyclone Impact layer, applied to each lemur species range. These summary statistics represent various representations of the overall level of influence of cyclones on each lemur species range derived from the Cyclone Impact model.

We would expect our measure of cyclone impact on a lemur range to be positively spatially correlated (i.e., clustered). The value of Cyclone Impact for grid cell and for each lemur range is likely to be similar in cells and ranges that are closer together. Therefore, we investigated spatial autocorrelation, a measure of spatial clustering or dispersion, in the Cyclone Impact results in two ways. First, a test of overall spatial autocorrelation of Cyclone Impact on lemur species range polygons was performed using Global Moran’s I with the Spatial Autocorrelation tool in ArcGIS Pro with the following parameters: lemur range feature layer as the input layer, Cyclone Impact Index as the input field, inverse distance to conceptualise the spatial relationship, Euclidean distance method, and we included row standardisation. Second, we investigated spatial autocorrelation of the clustering of Cyclone Impact among (i.e., local) lemur range polygons using the Anselin Local Moran’s I tool in ArcGIS pro using the same parameters as in the Global Moran’s I above. The Aneslin Local Moran’s I allows us to determine if there any clusters or outliers by comparing relative values of cyclone impact between species ranges. This tool tests for any lemur species ranges with significantly different values of cyclone impact when compared to other nearby species ranges.

### Lemur resilience score

We wanted to test lemur species’ ability to tolerate cyclones. Thus, we created an index that captured as many species traits as possible that are reported to be associated with cyclone resilience. Following [21] we applied a score-based method for 25 lemur species which were given a point for each cyclone resilient trait they possessed (see Table 1). To obtain these data, we surveyed published studies on wild lemurs for information on variables of interest (Appendix A). For all variables, we included studies from as many wild populations as possible that incorporated the variable of interest. This meant that for some variables we included one value and for others we took a mean of values. For diet, we set a criterion of studies being more than 9 months to ensure seasonal variation was incorporated (see supplementary data file for study list). For binary variables this included:

1. Energy conservation: any behaviours associated with energy conservation were given a point. This included torpor and hibernation.
2. Habitat use: species that are not strictly arboreal, using the ground for any key behaviours (i.e. foraging, grooming, sunbathing). Species that are not strictly arboreal and spend significant time out of the canopy were given a point.
3. Group size: Due to group size often being reported as a range, we treated this as a binary variable where groups less than 5 were considered small and those more than 5 considered large. Species with mean group sizes less than 5 were given a point. The value of 5 was used as it was a natural cut off point in our data where species typically had 2–5 members or greater than 10.

**Table 1:** Species resilience scores for 26 species of lemurs for which data on each resilience variable were available.

Species	Energy Conserving Behaviours	Habitat Use	Group Size	Fruit in the Diet	Home Range Size	Body Mass	Geographic range	Resilience Score
*Avahi laniger* (Eastern woolly lemur)	0	1	0	1	0	0	East	2
*Avahi meridionalis* (Southern woolly lemur)	0	1	0	1	0	1	East	3
*Avahi occidentalis* (Lorenz Von Liburnau’s woolly lemur)	0	1	0	1	0	1	West	3
*Cheirogaleus major* (Geoffroyi’s dwarf lemur)	1	1	1	0	0	1	East	4
*Chirogaleus medius* (Fat-tailed dwarf lemur)	1	1	1	0	0	1	West	4
*Daubentonia madagascariensis* (Aye-aye)	0	1	1	1	0	0	East and West	3
*Eulemur cinereiceps* (White-collared lemur)	0	0	0	0	1	0	East	1
*Eulemur collaris* (Collared brown lemur)	0	0	0	0	1	0	East	1
*Eulemur coronatus* (Crowned lemur)	0	0	0	0	0	0	East and West	0
*Eulemur flavifrons* (Blue-eyed black lemur)	0	0	0	0	0	0	West	0
*Eulemur fulvus* (Brown lemur)	0	0	0	0	0	0	East and West	0
*Eulemur macaco* (Black lemur)	0	0	0	0	0	0	West	0
*Eulemur mongoz* (Mongoose lemur)	0	0	0	0	0	0	West	0
*Eulemur rubriventer* (Red-bellied lemur)	0	0	0	0	0	0	East	0
*Hapalemur aureus* (Golden bamboo lemur)	0	1	0	1	0	0	East	2
*Hapalemur griseus* (Eastern lesser bamboo lemur)	0	1	0	1	0	0	East and West	2
*Hapalemur meridionalis* (Rusty-grey lesser bamboo lemur)	0	0	1	1	1	0	East	3
*Indri indri* (Indri)	0	1	1	1	0	0	East	3
*Lemur catta* (Ring-tailed lemur)	0	1	0	0	0	0	West	1
*Microcebus griseorufus* (Grey-brown mouse lemur)	1	1	1	1	0	0	West	4
*Microcebus murinus* (Grey mouse lemur)	1	1	0	0	0	1	West	3
*Propithecus diadema* (Diademed sifaka)	0	1	1	0	0	0	East	2
*Propithecus edwardsi* (Milne-Edward’s sifaka)	0	0	0	0	1	0	East	1
*Propithecus verreauxi* (Verreaux’s sifaka)	0	1	1	1	0	0	West	3
*Varecia rubra* (Red ruffed lemur)	0	0	0	0	1	0	East	1
*Varecia variegata* (Black-and-white ruffed lemur)	0	0	0	0	0	0	East	0

Energy conserving behaviours: 1 = present 0 = absent; Habitat use: 1 = arboreal and terrestrial 0 = strictly arboreal; Group size: 1 = less than 5 individuals 0 = more than 5 individuals; Fruit in the diet 1 = less than 50% 0 = more than 50%; Home range 1 = greater than 10ha 0 = smaller than 10ha; Body mass 1 = less than 898 grams 0 = more than 898 grams

For continuous variables, this required that we split the data at a certain value to create binary variables for inclusion in our models. We chose the splitting point by visually inspecting the density distributions of these variables and by plotting loess curves of these variables against our cyclone impact scores. Splits were chosen visually where there were obvious divides in the distribution of these variables or were loess curves changed direction. In other words, we inspected the data for natural clustering and selected a division point where data clustered obviously on one side or the other of that value. This included:

1. Fruit in the diet: species whose diet was made up of less than 50% fruit annually were given a point. Fifty per cent was chosen as previous studies have shown the species who rely on fruit for approximately half of their diet are particularly vulnerable post-hurricane [19].
2. Home range: species whose home range was more than 10 ha were given a point.
3. Mean body weight: species that weighed, on average, less than 898 g were given a point.

### Individual variables

We also conducted a univariate analysis on specific outcome variables for each species thought to be influenced by Cyclone Impact, including body mass, percentage of fruit in each species’ diet and home range size. For these variables we used generalized least squares regression (GLS) using the gls function from the nlme v3.1 [26] package in R while considering phylogenetic independence and spatial autocorrelation (see below).

### Phylogenetic nonindependence and spatial autocorrelation among traits and Cyclone Impact

To determine whether any of the traits in our datasets showed a meaningful association with Cyclone Impact we needed to account for both phylogenetic non-independence and spatial autocorrelation. To achieve this, we employed the above generalized least square (GLS) analysis procedure which allows regression models to be fit to outcome variables while also correcting for the covariance expected owing to descent and spatial proximity [27]. We used a spatial distance matrix and phylogenetic distance matrix as input, and then used these to derive a joint covariance matrix, i.e., the amount of autocorrelation expected given certain weights of phylogenetic and spatial autocorrelation [27]. Specifically, this approach uses the *subplex* function from the R package *nloptr* [28] to find the maximum-likelihood estimates of the regression models coefficients over different weights for each class of autocorrelation. This provides estimates of the correlation coefficients for outcome and predictor variables, as well as the amount of and relative spatial and phylogenetic autocorrelation in the dataset, and the rate of decay of the spatial autocorrelation (amount of influence of spatial autocorrelation decrease over a unit of distance). The model assumes that the spatial autocorrelation follows a general correlation structure.

To obtain a phylogenetic covariance matrix, we downloaded one thousand trees for the superfamily Lemuroidea from the online repository VertLife.org. Each of these trees is a subset drawn from the posterior of Upham et al.’s [29] Bayesian node-dated complete-evidence mammal-wide phylogenetic analysis. From this sample of potential phylogenies, we extracted the maximum clade credibility tree (MCC tree), the tree with the overall highest support for its clades within the sample. This tree was then used to calculate a phylogenetic distance matrix. To account for spatial autocorrelation within each regression analysis we created a spatial proximity matrix of the Euclidian distances between the centroids of each species’ range.

Predictor variables included the average Cyclone Impact of the grid squares which intersected with each species’ geographic range and the geographic range of each species (ha). We used the average Cyclone Impact across the species range to avoid collinearity between our measure of impact and geographic range and fit each Cyclone Impact scale (impact^87.5km^, impact^175km^, impact^350km^) separately to the outcome variables along with geographic range. All predictor variables at all scales were transformed by the natural log (ln) because at the 87.5 km radius Cyclone Impact was shown to be distributed non-normally by the Shapiro-Wilk normality test (*p* < 0.01), as was geographic range (*p* < 0.01). For all outcome variables, we fit all Cyclone Impact scales with and without geographic range as well as geographic range-only and intercept-only models. We used the Akaike Information Criteria for small sample sizes to identify the best fitting model, and then ran log likelihood ratio tests to determine if this model was a better fit than an intercept only model [30]. For each of the best fitting models, residuals were plotted against fitted values to check for heteroskedasticity and quantile-quantile plots were used to determine if residuals were distributed normally.

### Model assumptions and limitations

The Cyclone Impact model makes some assumptions about the nature of cyclone size, intensity, and impact. These assumptions should be considered when interpretating the model and for any downstream applications. Noted assumptions are:

- The degree of impact between Saffir classes is assumed to be linear. The impact of a tropical storm is difficult to quantify. Any comparison between classes would require an in-depth analysis of characteristics such as slope, aspect, wind speed and direction, etc. Given that the resulting analysis of cyclone impact on lemur ranges shows clear variance across the ranges, this assumption does not appear to impact the viability of the model. It also ensures any estimates of cyclone impact are coarse. A conservative estimate prevents over-estimating cyclone impacts on any particular lemur species range.
- Range of impact from a storm was homogenized to an average of 150km for each storm. This is unlikely to represent real world events, as storms will vary in size within and between Saffir classes. More complex buffer operations or a kernel density analysis may provide extra resolution to the model. However, it is unlikely to add value in discerning differences between lemur species ranges and impacts of cyclones.

## Results

### Cyclone impact and spatial autocorrelation

**Fig 1:**
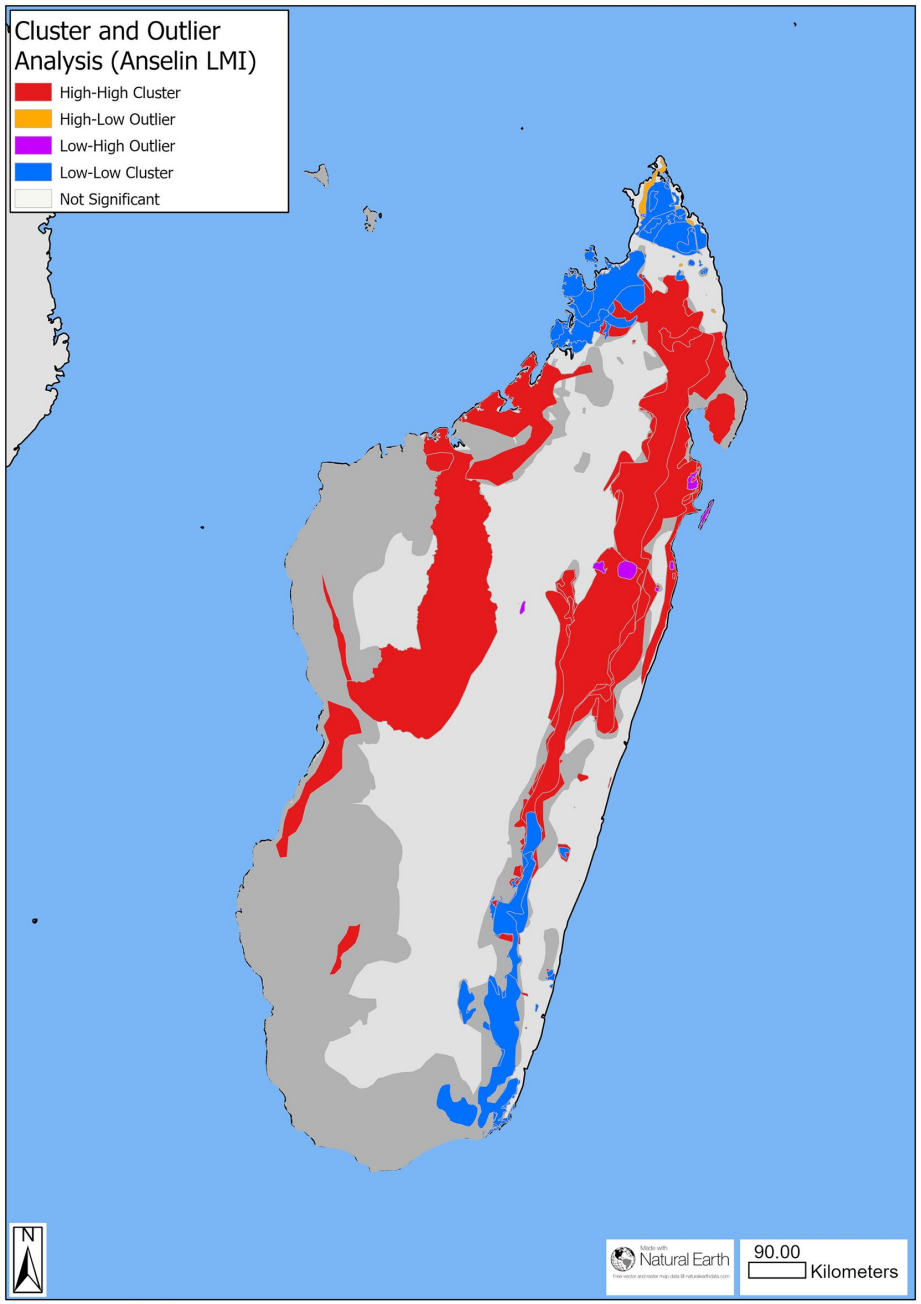
Anselin Local Moran’s I (LMI) cluster and outlier analysis (Anselin LMI). Clusters indicate areas of significant correlation between high or low Cyclone Impact on a lemur range size, whereas outlier ranges indicate significant inverse correlation between Cyclone Impact compared to surrounding lemur range size.

There were 3548 storms within 500 km of mainland Madagascar between 1963 and 2021 (S1a Fig). Cyclone Impact values for each grid square, a measure of the relative impact of cyclones between grid squares, ranged from 0–340 (S1b Fig). The cumulative Cyclone Impact on lemur species geographic ranges ranged from 11–11,367 (S1c Fig). Global Moran’s I analyses indicated that there was significant positive spatial autocorrelation of Cyclone Impact on lemur geographic range size (Moran’s I 0.303; p-value <0.001; S2 Fig), suggesting that lemur ranges of similar size were closer to each other. The Local Anselin Moran’s I indicated that there was significant spatial clustering in Cyclone Impact among lemur species with smaller ranges (Fig 1).

### Resilience score

We were able to obtain resilience scores for 26 species of lemur, ranging from zero to four points. The best fitting model was the one that included Cyclone Impact at 175 km scale and lemur geographic range size (Table 2). However, while range size was a significant predictor of resilience (range size, *β* = 0.149 ± 0.018, df = 23, t = 8.336, *p* < 0.001), Cyclone Impact was not (ln(impact^175km^), *β* = -0.374 ± 0.226, t = -1.651, df = 23, *p* = 0.112). According to a loglikelihood ratio test, this model was a significantly better predictor of the data than the intercept-only model (Likelihood ratio test, *χ*2 = 25.859, *p* < 0.001) and the model which only included range size (Likelihood ratio test, *χ*2 = 6.141, *p* < 0.013). Overall autocorrelation explained ~73% of the variance in the model, with ~75% of that attributed to phylogenetic autocorrelation. The decay in spatial autocorrelation was ~43% per unit distance. Thus, while including Cyclone Impact improves overall model performance compared to the model that only includes range size, the predictor itself was not significant, suggesting that the extra variance explained by the inclusion of cCyclone Impact is minimal. When fit without accounting for phylogenetic or spatial autocorrelation, the best fitting GLS model was the intercept-only model.

**Table 2:** Model fit indices for analysis of cyclone impact on lemur resilience score.

Model	AICc	δ	W	rLL	AC	phylo.	decay
~ impact^175km^ + range	46.275	0.000	0.669	1.000	0.731	0.749	0.433
~ impact^87.5km^ + range	49.348	3.073	0.144	0.215	0.810	0.808	0.498
~ range	49.847	3.572	0.112	0.168	0.886	0.863	0.135
~ impact^350km^ + range	50.884	4.609	0.067	0.100	0.715	0.721	0.484
~ impact^87.5km^	55.265	8.990	0.007	0.011	0.793	0.771	0.584
~ impact^175km^	62.963	16.688	0.000	0.000	0.770	0.736	0.615
~ impact^350km^	65.912	19.637	0.000	0.000	0.650	0.617	0.557
~ 1	67.210	20.935	0.000	0.000	0.572	0.557	0.456

Table includes the model structure, AICc, the delta (δ), model weight (*w*), relative likelihood (rLL), the amount of autocorrelation in the data (AC), the proportion of the autocorrelation that is phylogenetic (phylo) and the rate of decay in spatial autocorrelation.

### Individual variables

When variables were considered individually, we found that none of the binary variables (e.g., energy conserving, habitat use, or group size) was significantly predicted by Cyclone Impact at any scale and none of the models was a significantly better fit to the data than the intercept-only model.

Continuous variable analysis (e.g., body mass, fruit consumption in diet, home range size) had varying results. For body mass (Table 3), the best fitting model included both range size and Cyclone Impact at the 87.5 km scale (AICc = 119.046). Under this model there was a significant negative relationship between ln(Cyclone Impact^87.5km^) and ln(body weight) (*β* = -0. 184 ± 0.043, df = 61, t = -4.227, *p* < 0.001) and ln(Cyclone Impact^87.5km^) and ln(geographic range) (*β* = -0. 067 ± 0.029, df = 61, t = -2.267, *p* = 0.027). Thus, for every 100% increase in Cyclone Impact, we expect to see a 17% decline in body size, and for 100% increase in geographic range size, we expect to see a 6% decline in body size. Overall autocorrelation in the model was ~ 99%, of which 20% was phylogenetic. The decay rate of spatial autocorrelation was 9% per unit distance. The best fitting model for body mass was a significantly better predictor of the data than the intercept-only model (Likelihood ratio test, *χ*2 = 22.327, *p* < 0.001). When GLS were fit without accounting for spatial or phylogenetic autocorrelation, the best fitting model was the intercept-only model (AICc = 184.137), suggesting that the interactions detected above are masked by autocorrelation.

**Table 3:** Model fit indices for analysis of cyclone impact’s influence on lemur body mass.

Model	AICc	delta	W	rLL	AC	phylo.	decay
Body mass ~ impact^87.5km^ + range	119.046	0.000	0.428	1.000	0.990	0.980	0.093
Body mass ~ impact^175km^ + range	119.249	0.203	0.387	0.903	0.990	0.979	0.091
Body mass ~ impact^87.5km^	120.721	1.675	0.185	0.433	0.990	0.976	0.072
Body mass ~ impact^175km^	134.131	15.085	0.000	0.001	0.990	0.996	0.077
Body mass ~ impact^350km^ + range	135.550	16.504	0.000	0.000	0.987	0.993	0.091
Body mass ~ impact^350km^	135.933	16.888	0.000	0.000	0.986	0.996	0.079
Body mass ~ 1	137.192	18.146	0.000	0.000	0.980	0.993	0.083

Table includes the model structure, AICc, delta, model weight (w), relative likelihood (rLL), the amount of autocorrelation in the data (AC), the proportion of the autocorrelation that is phylogenetic (phylo) and the rate of decay in spatial autocorrelation.

For fruit consumption, the best fitting model was that which included only the natural log of Cyclone Impact at the 175 km scale as a predictor variable (Table 4; AICc = 303.356). Under this model there was a significant positive relationship between Cyclone Impact and the mean percentage of fruit in the diet (*β* = 6.177± 0.674, df = 33, t = 9.171, *p* < 0.001), thus for 100% increase in Cyclone Impact, we expect to see a ~6% increase in fruit as a percentage of diet. Overall autocorrelation in the dataset was ~99% with ~21% being phylogenetic autocorrelation. The decay rate of spatial autocorrelation was 3% per unit distance. This model was a significant improvement over the intercept-only model (Likelihood ratio test; 13.4, *p* < 0.001). When GLS regressions were fit without correcting for spatial and phylogenetic autocorrelation, fruit in the diet was best explained by the model that included Cyclone Impact at the 175 km scale and geographic range size as predictors. However, neither predictor variable was significant (Cyclone Impact^175km^, *β* = -14.5 ± 7.427, df = 32, t = 1.052, *p* = 0.059; geographic range size, *β* = -0.678 ± 1.993 df = 32, t = -0.34, *p* = 0.736). This model was a significantly better fit than the intercept-only model (Likelihood ratio test, 12.79, *p* = 0.002).

**Table 4:** Model fit indices for analysis of cyclone impact’s influence on lemur fruit consumption.

Model	AICc	delta	W	rLL	AC	phylo.	decay
Mean fruit ~ impact^175km^	303.356	0.000	0.775	1.000	0.990	0.794	0.032
Mean fruit ~ impact^87.5km^ + range	305.903	2.547	0.217	0.280	0.990	0.794	0.032
Mean fruit ~ 1	314.357	11.001	0.003	0.004	0.990	0.941	0.006
Mean fruit ~ impact^87.5km^	315.827	12.471	0.002	0.002	0.990	0.935	0.008
Mean fruit ~ impact^350km^	316.006	12.650	0.001	0.002	0.990	0.933	0.007
Mean fruit ~ range	316.693	13.338	0.001	0.001	0.938	0.989	0.808
Mean fruit ~ impact^175km^ + range	317.979	14.624	0.001	0.001	0.990	0.938	0.008
Mean fruit ~ impact^350km^ + range	318.488	15.132	0.000	0.001	0.990	0.937	0.007

Table includes the model structure, AICc, delta, model weight (*w*), relative likelihood (rLL), the amount of autocorrelation in the data (AC), the proportion of the autocorrelation that is phylogenetic (phylo) and the rate of decay in spatial autocorrelation.

The model that best explained the distribution of home range size data included the Cyclone Impact at the 350 km scale and geographic range size (Table 5; AICc = 181.542). Under this model there was a significant association between home range size and geographic range size (*β* = -0.558 ± 0.195, df = 24, t = -2.864, *p* = 0.007) but no significant association between home range size and any measure of Cyclone Impact (*β* = -1.645 ± 0.957, df = 34, t = -1.718, *p* = 0.096). This model was a significantly better fit than the intercept-only model (Likelihood ratio test, *χ*2 = 10.3, *p* = 0.006). Autocorrelation explained ~ 70% of the variance in the data of which 11% was phylogenetic. The decay rate of spatial autocorrelation was <1% per unit distance. When GLS were fit without correcting for autocorrelation, the best fitting model for home range size was also that which included Cyclone Impact at 350 km scale and geographic range size (AICc = 184.977). As with the autocorrelation analysis, the only significant association was a negative one between home range size and geographic range size (*β* = -4.049 ± 0.199 df = 34, t = -2.062, *p* = 0.047) and while the relationship between home range size and Cyclone Impact was negative it was insignificant (*β* = -1.1348 ± 1.127, df = 34, t = -1.2, *p* = 0.241). This model was found to be a significantly better fit than the intercept-only model (Likelihood ratio test; 6.95, *p* = 0.031).

**Table 5:** Model fit indices for analysis of cyclone impact’s influence on lemur home range size.

Model	AICc	Delta (δ)	W	rLL	AC	phylo.	decay
Home range ~ impact^350km^ + range	181.542	0.000	0.391	1.000	0.697	0.886	0.008
Home range ~ range	182.029	0.487	0.306	0.784	0.600	0.975	0.375
Home range ~ impact^175km^+ range	183.882	2.341	0.121	0.310	0.651	0.914	0.002
Home range ~ impact^87.5km^ + range	184.439	2.897	0.092	0.235	0.658	0.882	0.002
Home range	186.129	4.587	0.039	0.101	0.573	0.678	0.007
Home range ~ 1	186.915	5.374	0.027	0.068	0.503	0.000	0.001
Home range ~ impact^175km^	188.271	6.729	0.014	0.035	0.511	0.613	0.000
Home range ~ impact^87.5km^	188.953	7.411	0.010	0.025	0.514	0.478	0.002

Table includes the model structure, AICc, δ, model weight (*w*), relative likelihood (rLL), the amount of autocorrelation in the data (AC), the proportion of the autocorrelation that is phylogenetic (phylo) and the rate of decay in spatial autocorrelation.

## Discussion

This study is the first to use a comparative approach to investigate a quantitative link between cyclone activity across Madagascar and the presence of resilience traits in lemur species, while controlling for phylogenetic and spatial autocorrelation. We found no strong relationship between Cyclone Impact and resilience score for the species for which we obtained enough data (N = 26). We also found no relationship between Cyclone Impact and energy conserving behaviours, habitat use, group size and home range size. We did find an association between body size and Cyclone Impact with smaller species being associated with higher impact cyclone zones. While we also found an association with fruit, this was the opposite of what we expected, with heavy Cyclone Impact associated with higher fruit consumption.

The lack of clear effect of cyclone activity on our cumulative index of lemur resilience traits can be explained by a few different possibilities. First, it could be that recent cyclone activity is a poor proxy for the historic effect of cyclone activity across Madagascar and a deeper investigation into historic cyclone activity is needed to capture its long-term effect on adaptation in these species. However, this may not be possible due to the limited number of years for which cyclone data has been accurately recorded. Indeed, Nash et al. [31] suggested that fewer tropical cyclones made landfall during the 19^th^ century than in more recent years. The tropical storm data modelled over Madagascar in our study only covers the last 58 years as no longer term data are available. The assumption that cyclone impacts have been consistent over an evolutionary timescale is thus an unavoidable limitation of this work. We also found that resilience scores in our sample are phylogenetically clustered. *Eulemur*, in particular was found to have nearly identical resilience scores with five species having scores of 0 and three having scores of 1; despite these species living in a variety of habitat types and their ranges having quite different cyclone impact scores.

While there are several cyclones that make landfall in the west, the majority follow prevailing weather patterns and make landfall in the northeast and east. These prevailing weather patterns create very different rainfall gradients from high east to low west [32]. Meanwhile there is ecogeographic size variation in lemurs with the largest lemurs occurring in the central highlands (extinct species) and the rest decreasing in size in the humid east, followed by the arid west, then the arid northwest, then the arid south [33]. Lemurs are also more frugivorous in the humid east than in the arid west [34]. While cyclones may not be driving these variations, they may in some cases exacerbate the patterns. For example, across sifaka species body size was found to be associated with seasonality being positively associated with rainfall in the east and negatively associated with length of dry season in the west [35]. Cyclones contribute more to greater seasonality in the west than in the east which is more accustomed to higher levels of rainfall throughout the year. Although cyclones (hurricanes) have been shown to severely reduce fruit availability in other regions [17], perhaps in eastern Madagascar, their high frequency is partly responsible for driving increased fruit availability.

Secondly, it could be that the features thought to be adaptations to regular cyclone activity have in fact evolved in species in Madagascar for a different reason. It has been previously suggested that the intense yet predictable seasonality that exists in Madagascar has led to the evolution of behavioural flexibility that would also allow resilience to unpredictable cyclone activity [9]. Similarly, high levels of inactivity and energy conserving behaviours, while allowing animals to survive better after a cyclone, could have evolved as a means to improve digestion in species active all throughout the day and night [30]. This is supported by the fact that Madagascar has a notably high number of such cathemeral species, including many species in the Lemuridae [36]. That said, within lemurs, only nocturnal primates have evolved the ability to enter periods of torpor or hibernation and even this varies between species [38]. While torpor in *Cheirogaleus* spp. is considered to be a response to seasonal food scarcity, it could also reflect that diurnal species are using behavioural adaptations, including sunbathing and huddling, to cope with seasonal differences while nocturnal species developed physiological strategies [37]. It may also be that species are adapted to very distinct environments across Madagascar, and variation in their ecological traits is more associated with general conditions including precipitation and temperature differences than by extreme weather events and associated disturbances.

Dewar and Richard [38] have also suggested that it is the high levels of interannual rainfall across the entire island and an associated unpredictability in fruit production that can explain a reliance on folivory in Malagasy species compared to mainland counterparts. These explanations fit well with our initial hypothesis that frugivory would be negatively associated with cyclone activity; in other words where more cyclones occur species rely on less fruit. Frugivory was predicted to decrease in high cyclone zones because cyclones and hurricanes are known to alter plant productivity with energy allotment moving from reproduction to survival in plants that are heavily damaged. This has delayed fruit production after some severe windstorms for over a year [17]. Ameca et al. [21] found that diet specialisation, including heavy reliance on fruit, clustered with habitat specialisation in species highly vulnerable to hurricanes, supporting the suggestion that species with more generalist diets are more resilient to living in a hurricane zone. Contrary to this expectation, we found a significant positive relationship between cyclone activity and fruit consumption with frugivory increasing in more heavily impacted cyclone zones. As the unique environment of Madagascar is hit by multiple cyclones each year, habitats are essentially undergoing a multitude of small-scale disturbances in the form of tree-fall canopy gaps [39–41]. These gaps may increase fruit production and in a previous study were determined to be areas of high food abundance for frugivorous species including the red-ruffed lemur (*Varecia rubra)* and The white headed lemur (*Eulemur albifrons (cite))*. Additionally, following Cyclone Gretelle in 1997, the white-collared lemur (*Eulemur cinereiceps)* initially showed a decline in population numbers and group size, however, at 10 years post hurricane population numbers were the same as pre-cyclone, despite reductions in most vegetative measures [40]. As white-collared lemurs are largely frugivorous, this supports the idea that frugivorous species in Madagascar can be resilient to cyclone activity, possibly due to their fast reproductive rates compared to their folivorous counterparts [42]. Therefore, in Madagascar, fruit consumption may actually be a strategy that takes advantage of tree fall gaps associated with multiple cyclone events, suggesting that the small-scale disturbances may be having a greater selective pressure. Higher fruit tree species diversity in the north and east [43] mirror our results that found higher cyclone impact scores in these regions. This result may also reflect that while cyclones do traverse the entire island, they do occur more in the east which has contributed to the overall different biomes across the island—with wetter conditions being more prevalent in the east tapering to drier conditions in the west. Frugivory may thus be an adaptation to the wetter rainforests of the east, where plant diversity is higher as is overall fruit availability despite the frequent cyclone exposures. That said, this does not explain the generalised lack of frugivorous species on Madagascar, so while these small canopy gaps may be providing enough fruit to slightly increase fruit intake and allow for increased reproduction [39–41], our results suggest they are not having an overall impact on how many frugivorous species can co-exist on the island.

Our results also revealed a relationship between cyclone score and body size, with a higher cyclone impact score being associated with smaller species. This supports previous work as larger primates need more food and space and generally have slower life histories [44–48], they are predicted to be less resilient and show higher mortality following severe habitat disturbance [49]. Lemurs are thought to generally show the same trend, although a recent study found that within extant and extinct lemurs, brain size is a better proxy for slow life history than body size for some species [50]. While our results support previous findings that smaller species are less vulnerable to cyclone damage, it is important to note that the largest extant lemur (Indri) are found in the Northeast of the island where there is heavy cyclone activity, indicating that larger species may also show relevant adaptations. That said, larger lemur species do still require more space and food requirements, supporting our finding that smaller species would be less vulnerable to cyclone damage.

As ours is the first study to try to show a link between resilience traits and cyclone occurrence and intensity using a comparative approach that corrects for spatial and phylogenetic autocorrelation and because we found mostly null results, we must consider that these traits are not related to cyclone activity and are perhaps better explained as prerequisite traits for surviving colonization events. It is thought that all lemur species, with the possible exception of the aye-aye, evolved from one common ancestor splitting as early as 60 and as late as 20 million years ago [51–53]. This could suggest that the tolerance to disturbance and resilience we see across modern-day lemurs was already present in that common ancestor. Recent evidence points to this common ancestor rafting to Madagascar from mainland Africa on floating islands, which simulations suggest would have taken over three weeks [54]. Additionally other mammal groups found in Madagascar also show energy conserving behaviours that could be explained as requisite for colonization via rafting [55]. It stands to reason that these original mammal species would have already had to possess many of the features associated with resilience to disturbance to survive the journey, thus it is possible that the lack of relationship between our resilience score and cyclone impact score is because lemurs all possess these traits from their last common ancestor.

### Study limitations and future directions

There are a few limitations to our study. The first is a lack of life history and behavioural ecology data for many lemur species. Therefore, we were limited to conducting our analysis on 26 well-studied species. Although we tried to include species across lemur families, a larger sample size would have provided more variation as our current sample did not have any representation from Lepilemuridae and only included 26 of 106 species and 9 of 15 genera. Secondly, because the size and potential impact of a cyclone on vegetation (and hence lemur habitat) is poorly understood, we had to make assumptions that should be considered when interpreting the model and for any downstream applications. First, we assumed that the degree of impact between Saffir classes is linear, but the impact of a tropical storm is difficult to quantify. Any comparison between classes would require an in-depth analysis of characteristics such as slope, aspect, wind speed and direction, which was not possible for this study. However, given that the resulting analysis of cyclone activity on lemur ranges shows clear variance across the ranges, this assumption does not appear to impact the validity of the model. While these assumptions were unavoidable, we were able to consider the influence of cyclones at multiple scales as well as create a buffer that provided a conservative estimate of cyclone impacts on any given lemur species range. Both of these model additions ensured that we did not over-estimate Cyclone Impact, which could have biased our results. Finally, we acknowledge that classifying some continuous variables as binary, although necessary for the chosen analysis, may have lost some nuance in these variables. We are confident that using natural breaks in the data reflect a biologically relevant cut off point to split the data, but it is possible that this could have resulted in a loss of some of the variation that exists within and across species.

While our results did not support the assertation that differences in cyclone activity across Madagascar can explain variation in an index of traits expected to be associated with resilience, it is possible that our focus on Madagascar did not allow us to see a broader signal in all primates. A future study could thus create and apply a similar cyclone/hurricane impact score across different primate ranges globally, which could be compared to calculated resilience scores and individual traits in different regions of the globe. This analysis would allow much larger patterns to be revealed, which would help to better understand the impact of natural disasters on the evolution of primate traits and different species’ ability to be resilient in the face of a natural disturbance. Such a study would provide valuable information not only on the impact of natural disasters on the evolution of behavioural and morphological traits in primates, but would help to understand which species may be most vulnerable to future disasters by creating a global bank of resilience data associated with cyclone/hurricane risk from historical data. Given that under global climate change models, an increase in the frequency and/or intensity of cyclones and hurricanes is expected, this would allow us to better protect the most vulnerable and least resilient species.

## Supporting information

S1 Fig Cyclone impact model and historical storm tracks. a) Visualisation of the Cyclone Impact model (log scale). Grid squares with a null value were automatically excluded from the generated feature layer. Colour graduations are presented on a logarithmic scale to visually represent the data. b) Cyclone Impact model (log scale) with historical storm tracks by category (Saffir Class). c) cyclone impact on lemur ranges.
(TIF)

S2 Fig Global Moran’s I measure of spatial autocorrelation on cyclone impact on lemur ranges. Output of ArcPro Global Moran’s I calculations of spatial autocorrelation based on the cyclone impact on each lemur species range.
(TIF)

S1 File References and data used in the resilience score determination an modelling. (DOCX)

S1 Text (TXT)
