# Supplementary figures and images for: Can cyclone exposure explain behavioural and demographic variation among lemur species?
Source: PLoS One. 2024 Mar 27;19(3):e0300972. doi: 10.1371/journal.pone.0300972 (PMC10971772; doi:10.1371/journal.pone.0300972)

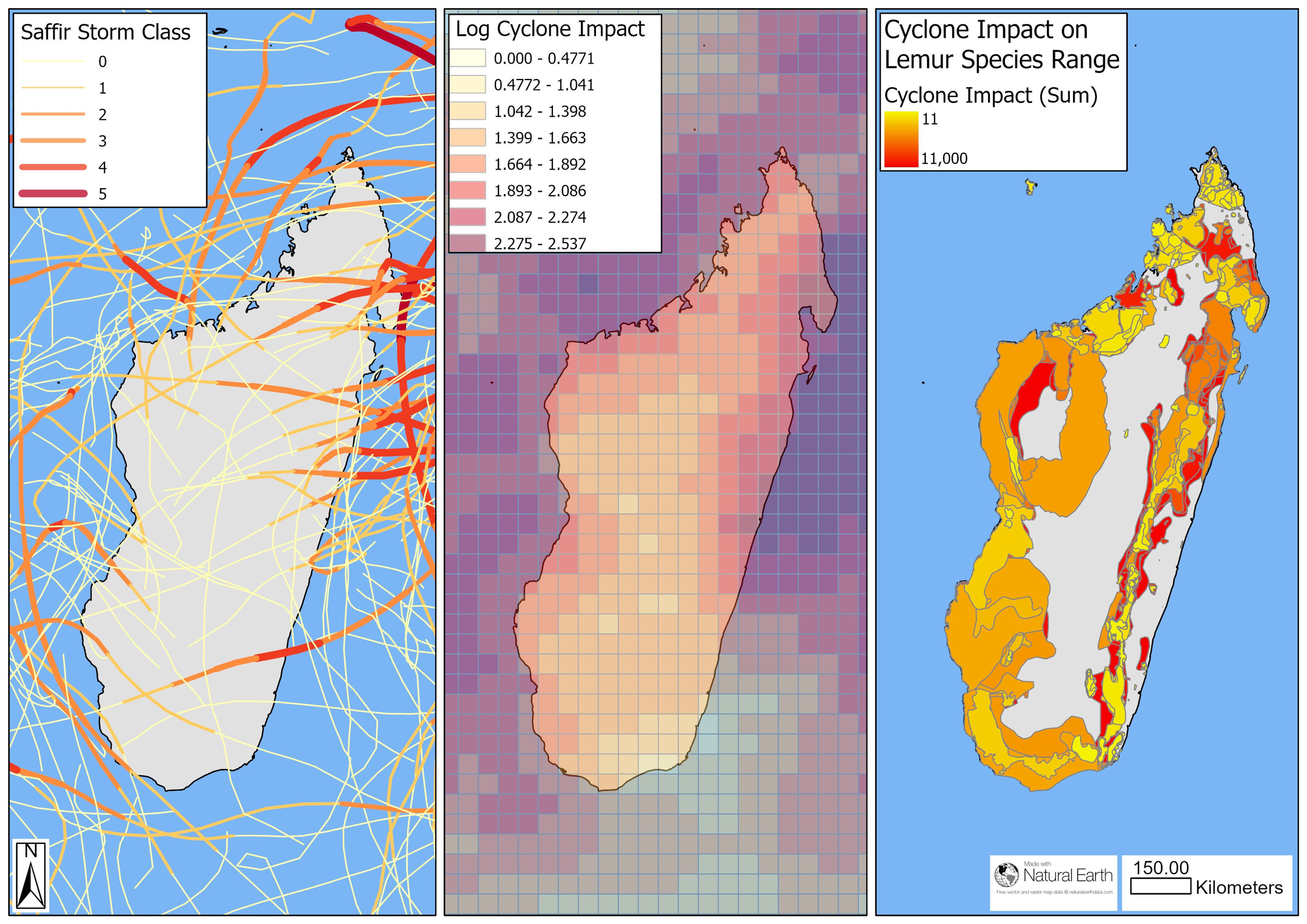

Supplement: S1 Fig — a) Visualisation of the Cyclone Impact model (log scale). Grid squares with a null value were automatically excluded from the generated feature layer. Colour graduations are presented on a logarithmic scale to visually represent the data. b) Cyclone Impact model (log scale) with historical storm tracks by category (Saffir Class). c) cyclone impact on lemur ranges. (TIF) [file pone.0300972.s001.tif]

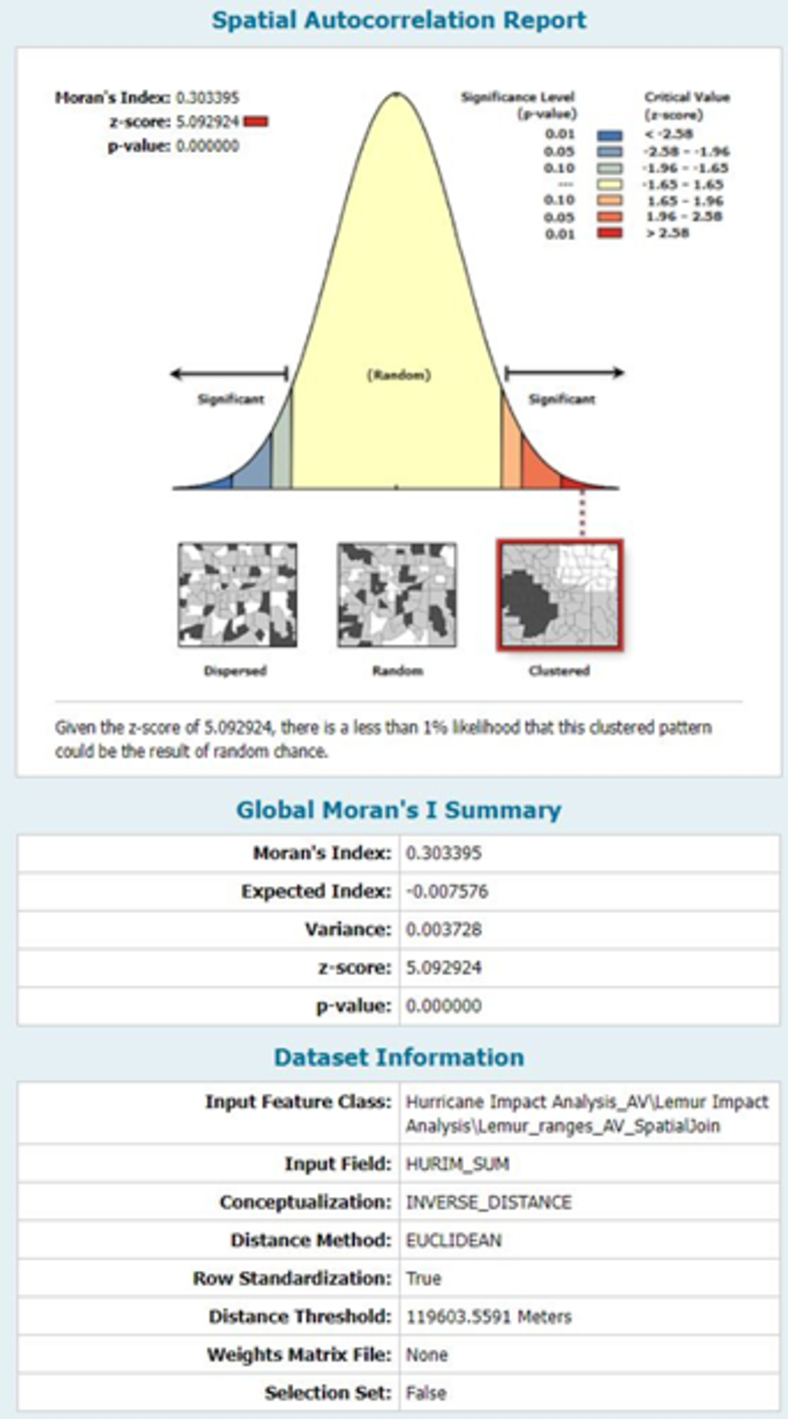

Supplement: S2 Fig — Output of ArcPro Global Moran’s I calculations of spatial autocorrelation based on the cyclone impact on each lemur species range. (TIF) [file pone.0300972.s002.tif]
