# Supplementary material for: Can cyclone exposure explain behavioural and demographic variation among lemur species?
Source: PLoS One. 2024 Mar 27;19(3):e0300972. doi: 10.1371/journal.pone.0300972 (PMC10971772; doi:10.1371/journal.pone.0300972)
Supplement: S1 File — (DOCX) [file pone.0300972.s003.docx]

**Supplementary Material. Data and References used for resilience scores and modelling.**

| **Species** | **Energy Conserving Behaviours** | **Habitat Use** | **Group Size** | **Fruit in the Diet** | **Home Range Size** | **Body Mass** |
| --- | --- | --- | --- | --- | --- | --- |
| *Avahi laniger* | No | Arboreal (Harcourt 1991) | 2-5 (Thalman & Geisman 2000) | <5% fruit (Harcourt 1991) | 1 – 4 ha (Harcourt 1991) | 1000-1300g (Zarmandy et al. 2006) |
| *Avahi meridionalis* | No | Arboreal (Norscia et al. 2011) | 2-5 (Norscia*,*2008) | >5% fruit (Norscia et al. 2011) | 2.0-3.5ha (Norscia*,*2008) | 1200g (Zaramody et al. 2006) |
| *Avahi occidentalis* | No | Foraging on ground (Schmidt 2010; Thalmann 2001) | 2-5 (Thalmann 2001) | 26% fruit (Thalmann 2001) | 1-2 ha (Warren & Crompton 1998)  1.39ha (Thalmann 2001) | 708g (Warren & Crompton 1998) |
| *Cheirogaleus major* | Hibernation (Wright & Martin 1995) | Ground burrows (Eppley et al 2011) | 2-5 (Lahann 2008) | 68% fruit (Lahmann 2007) | 3.3 – 4.8 ha (Lahann 2008)  4.4 ha (Lahann 2007) | 350-450g (Wright & Martin 1995; Mittermeier et a. 2006) |
| *Cheirogaleus medius* | Torpor (Dausmann et al 2004) | Ground burrows (Eppley et al 2011) | 2-5 (Fietz 1999) | 63% fruit (Lahann 2007) | 1.05-2.36 ha (Mullen 1989) 1.56-4.68 (Fietz 1999) | 300-480g (Lemelin & Schmitt 2004; Lahmann & Dausmann 2011) |
| *Daubentonia madagascariensis* | No | Foraging on ground (Sefczek et al. 2020) | Solitary (Sterling, 1993; Sterling & McCreless 2006) | <50% fruit (Randimbiharinirina 2018; Roen, 2014) | 0.765-2.586 ha (Sefczek et al. 2020) | 2050 – 3000g (Feistner & Sterling 1995) |
| *Eulemur cinereiceps* | No | Arboreal (Johnson 2002) | 5-17 (Johnson 2002)  Donati & Bollen 2007) | 66-95% (Johnson 2002)  67% (Ralainasolo et al. 2008)  93% (Andriamaharoa et al. 2020) | 33.5-64.3ha (Johnson 2002) | 2070 – 2290g (Johnson et al 2005) |
| *Eulemur collaris* | No | Arboreal (Eppley et al. 2017) | 7 (Kappelar & Fichtel 2016)  8-13 (Donati et al. 2007) | 66% (Donati et al. (2007) | 962ha (Eppley et al. 2017) | 2150g (Balestri et al 2015) |
| *Eulemur coronatus* | Torpor (Schmid et al. 2000; Schwab 2000; Schwab & Ganzhorn) | Arboreal (Chen et al. 2015) | 2.8-4.5 (Rakotondrina et al. 2023) | 92% (Chen et al. 2015) | 10-15ha (Freed 1996) | 1177g (Terranova & Coffman 1997) |
| *Euleumr flavifrons* | No | Forage on ground (Nunn et al. 2018 ) | 7.5 (Kappelar & Fichtel 2016)  7.4-8.6 (Volampeno et al. 2011a)  8.5 (Volampeno et al. 2011b) | 73% (Volampeno et al. 2011b) | 7.5 ha (Volampeno et al. 2011a) | 1793g (Terraova & Coffman 1997) |
| *Eulemur fulvus* | No | Arboreal (Sato et al. 2014) | 6.9 (mean; Ralison et al. 2010)  8.46 (mean; Keppelar & Fichtel 2016) | 67.6 % (Sato et al. 2014) | 39 ha (Sato 2013) | 2450-3000g (Gordon et al. 2016) |
| *Eulemur macaco* | No | Arboreal (Simmen et al. 2007) | 18.2 (Bayart & Simmen 2005)  11.7 (Kappelar & Fichtel 2016) | 93% (Simmen et al. 2007) | 5-5.5 (mean; Colquhoun 1993) | 2200 – 2350g (Gordon et al. 2016) |
| *Eulemur mongoz* | No | Arboreal (Curtis & Zaramody) | 2.8 (Kappelar & Fichtel 2016)  3 (Curtis & Zaramody) | 65% (Curtis 2003) | 2.8-2.9 ha (Curtis & Zaramody) | 1481g (Terranova & Coffman 1997) |
| *Eulemur rubriventer* | No | Arboreal (Overdorff 1993) | 2.8 (Kappelar & Fichtel 2016) | 80.6 (Overdorff 1993) | 10-14 ha (Overdorff 1993) | 1956g (Terranova & Coffman 1997) |
| *Hapalemur aureus* | No | Arboreal (Tan 1999) | 2-5 (Tan 1999) | < 5% fruit (Tan 1999) | Overdorff et al. 1997)80ha (Tan 1999) | 1600g (Glander et al) |
| *Hapalemur griseus* | No | Arboreal (Tan 1999) | 1-4 (Tan 1999) | <15% fruit (Tan 1999; Overdorff et al. 1997; Grassi 2002) | 15 ha (Tan 1999) | 956g (Glander 1992) |
| *Hapalemur meridionalis* | No | Forage terrestrially (Eppley 2011; 2015) | 5.5 (Eppley et al. 2015) | 18.58% (Eppley et al. 2015) | 17-18ha (Eppley et al. 2016) | 1070g (Eppley et al 2011; 2015) |
| *Indri indri* | No | Forage terrestrially (Irwin et al 2007; Pollock 1975) | 2-5 (Bonadonna et al. 2017)  3-5 (Pollock 1975)  2-4 (Correa et al. 2021) | 5.5% fruit (Britt et al. 2002)  16% fruit (Powzyk & Mowry 2003) | 27ha (Glessner & Britt 2005)  13.7 ha (Bonadonna et al. 2017) | 950g-  1600g (Glander & Powzyk 1998) |
| *Lemur Catta* | No | 15-30% time terrestrial (Mittermeier et al. 2010) | 13.2 mean (Pride et al. 2005)  9.2-11.5 (Gould & Sussman 2003)  6-17 (Kelley 2013) | 48-70%; 45-85% monthly (Gould et al. 2014) | 8-132 ha (Kelley 2013) | 2200-2700g (Koyama et al. 2008) |
| *Microcebus griseorufus* | Torpor (Kobbe et al. 2011) | Forage on ground in dry season (Rodriguez et al. 2015) | Solitary (Kobbe et al. 2011) | 45% (Bohr et al. 2011)  20% (Genin et al. 2008) | <1ha (Bohr et al. 2011)  1ha (Genin 2008) | 39-50g (Bohr et al. 2011)  51-59g (Genin 2008)  42-83g (Kobbe et al. 2011)  63g (Rasolooarison et al. 2000) |
| *Microcebus murinus* | Torpor (Ganzhorn & Schmid 1998; Schmid 2007; Schmid & Kappelar 1998; Schulke & Ostner 2007) | Travel on ground (Rahlfs & Fichtel 2010) | Solitary (Perret &Aujard 2001) | 63% (Lahann 2007)  0-80% monthly (Thoren et al. 2011) | 1-5ha (Radespiel 2000) | 40-100g (Perret & Aujard 2001)  50.1 – 105.6g (Lahann et al. 2006) |
| *Propithecus diadema* | No | Forage on the ground (Irwin 2008) | 4-6 (Irwin 2008)  2-6 (Irwin 2007) | 42% (Powzyk & Mowry 2003)  24% (Irwin et al. 2007)  15-19% (Lehmann & Mayer 2007) | 21-83 ha (Irwin 2008) | 5000 – 7000g (Gordon et al. 2013) |
| *Propithecus edwardsi* | No | Arboreal (Gould & Sauther 2006) | 4.8 (Wright 1998) | 20-32% (Hemingway)  21-30% (Matos et al. 2022) | 18-46ha (Gerber et al. 2012) | 5800g (Wright 1998) |
| *Propithecus verreauxi* | No | Foraging on ground (Brockman et al. 2008) | Mean 4-6 (Benadi et al. 2008)  Mean 5-6 (Richard 1985) | Approximately 25% (Lewis and Kappelar 2005)  Approximately 30% (Koch et al. 2017) | 6-10ha (Benadi et al. 2008)  1.0-8.5 ha (Richard 1985) | 3000-4000g (Gordon et al 2013)  3019-3574g (Lewis and Kappelar 2005) |
| *Varecia rubra* | No | Arboreal (Vasey 2004) | 5 (Rigamonti 1993)  2-6 (Martinez & Razafindratsima) | 80-100% (Vasey 2004)  74% (Rigamonti 1993)  61% (Martinez & Razafindratsima) | 23.3 – 25.8ha (Rigamonti 1993)  57ha (Vasey 2007) | 3740-4720g (Hartstone-Rose & Perry 2011) |
| *Varecia variegata* | No | Arboreal (Perreira et al. 1988; Vasey 1997) | 7 (Balko & Underwood 2005)  7 (Pereira et al. 1988)  3 (Balko et al. 2005) | 92% (Britt 2000)  69.4% (White et al. 1995) | 57.7ha (Vasey 1997) | 2750g-3760g (Ratsimbazafy 2002) |

*No references are listed when energy conserving behaviour is no as the absence of a behaviour is often not documented. Rather, for species who at the time of publication had no accounts of hibernation or torpor, they were given a No and a score of 0.

**Reference list**

##### Andriamaharoa H, Birkinshaw C, Reza L. Day-time feeding ecology of Eulemur cinereiceps in the Agnalazaha Forest, Mahabo-Mananivo, Madagascar. *Madagascar Conservation & Development*. 2010; *5:* doi: 10.4314/mcd.v5i1.57341

Balestri M, Barresi M, Campera M, Serra V, Ramanamanjato JB, Heistermann M, Donati G. Habitat degredation and seasonality affect physiolocial stress levels of Eulemur collaris in littoral forest fragments. PlosOne. 2014; [doi: 10.1371/journal.pone.0107698](https://doi.org/10.1371/journal.pone.0107698)

Balko EA, Underwood B (2005), Effects of forest structure and composition on food availability for *Varecia variegata* at Ranomafana National Park, Madagascar. Am. J. Primatol. 2005; 66: 45-70. [doi: 10.1002/ajp.20127](https://doi.org/10.1002/ajp.20127)

Bayart F, Simmen B. (2005). Demography, range use, and behavior in black lemurs (Eulemur macaco macaco) at Ampasikely, northwest Madagascar. *Am J Primatol. 2005*; *67*: 299-312.

Benadi G, Fichtel C, Kappelar P. Intergroup relations and home range use in Verraux’s sifaka (Propithecus verrauxi). Am J Primatol. 2008: 70: 956-965.

Bohr YEMB, Giertz P, Ratovonamana YR, Ganzhorn JU. (2011). Gray-brown mouse lemurs (Microcebus griseorufus) as an example of distributional constraints through increasing desertification. *Int Journal Primatol. 2011*; *32*: 901-913.

Britt, A. Diet and feeding behaviour of the black-and-white ruffed lemur (Varecia variegata variegata) in the Betampona Reserve, eastern Madagascar. *Folia Primatol*. 2000; 71: 133-141.

Brockman DK, Godfrey, LR, Dollar, LJ*. et al.* Evidence of Invasive *Felis silvestris* Predation on *Propithecus verreauxi* at Beza Mahafaly Special Reserve, Madagascar. *Int J Primatol*. 2008; 29,=; 135–152. [doi: 10.1007/s10764-007-9145-5](https://doi.org/10.1007/s10764-007-9145-5)

Chen KS, Li JQ, Rasoarahona J, Folega F, Manjaribe C. Diet and seed dispersal by Eulemur coronatus (Gray, primates and Lemuridae) in the Amber Mountain National Park, Madagascar. *Int J Biol. 2015;*  *7*:4.

Colquhoun IC. (1993). The socioecology of Eulemur macaco: a preliminary report. In Kappelar PM, Ganzhorn JU. editors. *Lemur social systems and their ecological basis*. Boston, MA: Springer US. Pp:11-23.

Curtis DJ. Diet and nutrition in wild mongoose lemurs (*Eulemur mongoz*) and their implications for the evolution of female dominance and small group size in lemurs. *Am J Phys Anthropol. 2004; 124*: 234–247.

Curtis DJ, Zaramody A. Group size, home range use, and seasonal variation in the ecology of Eulemur mongoz. Int J Primatol. 1998; 19: 811-835.

Dammhahn M, Kappeler PM. (2008). Comparative feeding ecology of sympatric Microcebus berthae and M. murinus. *Int J Primatol. 2008;* *29*: 1567-1589.

Dausmann KH, Glos J, Ganzhorn JU, Heldmaier G. Physiology: hibernation in a tropical primate. Nature. 2004 Jun 24;429(6994):825-6. doi: 10.1038/429825a. PMID: 15215852.

Donati G, Bollen A, Borgognini-Tarli SM, Ganzhorn JU. Feeding over the 24-h cycle: dietary flexibility of cathemeral collared lemurs (Eulemur collaris). *Behav Ecol Sociobiol. 2007;* *61*: 1237-1251.

Donati G, Kesch K, Ndremifidy K, Schmidt SL, Ramanamanjato JB, Borgognini-Tarli SM, Ganzhorn JU. Better few than hungry: flexible feeding ecology of collared lemurs *Eulemur collaris* in littoral forest fragments. PLoS One. 2011; *6*: e19807.

Eppley TM, Ganzhorn JU, Donati G. Latrine behaviour as a multimodal communicatory signal station in wild lemurs: the case of Hapalemur meridionalis. *An Behav*. 2015; 111; 7-67. doi: 10.1016/j.anbehav.2015.10.012.

Eppley TM, Balestri M, Campera M, Rabenantoandro J, Ramanamanjato JB, Randriatafika, F, Donati G. Ecological flexibility as measured by the use of pioneer and exotic plants by two lemurids: *Eulemur collaris* and *Hapalemur meridionalis*. Int J Primatol*. 2017;* *38*: 338-357.

Feistner ATC, Sterling EJ. Body mass and sexual dimorphism in the aye-aye *Daubentonia madagascariensis*. *Dodo*. 1995; 31: 73-76.

Fietz J. Monogamy as a rule rather than exception in nocturnal lemurs: the case of the fat‐tailed dwarf lemur, Cheirogaleus medius. *Ethology*. 1999; 105:255-72.

Fietz J, Ganzhorn JU. Feeding ecology of the hibernating primate *Cheirogaleus medius*: How does it get so fat? *Oecologia*. 1999; 121: 157–164.

Freed, B. Z., 1996: *A comparative ecological study of two sympatric species of primate, Lemur coronatus and Lemur fulvus sanfordi, in northern Madagascar*. Dissertation, Washington University, Saint Louis.

Ganzhorn JU, Schmid J. Different population dynamics of Microcebus murinus in primary and secondary deciduous dry forests of Madagascar. *Int J J J Primatol. 1998;* *19*: 785-796.

Génin F. Life in unpredictable environments: first investigation of the natural history of Microcebus griseorufus. *Int J Primatol. 2008; 29*: 303-321.

Glander KE, Powzyk. JA. Morphometries of wild Indri indri and *Propithecus diadema diadema*. *Folia Primatol*. 1998; 69(S1): 399.

Gordon AD, Johnson SE, Louis Jr EE. Females are the ecological sex: sex-specific body mass ecogeography in wild sifaka populations (Propithecus spp.). Am J Primatol. 2013; 151: 77-87.

Gordon AD, Johnson SE, Louis Jr EE. Environmental Correlates of Body Mass in True Lemurs (*Eulemur* spp.). Int J Primatol. 2016; 37: 89–108 doi: 10.1007/s10764-015-9874-9

Gould L, Sussman RW, Sauther, ML. Demographic and life-history patterns in a population of ring-tailed lemurs (*Lemur catta*) at Beza Mahafaly Reserve, Madagascar: A 15-year perspective. Am J Phys Anthropol. 2003; 120: 182-194. [doi: 10.1002/ajpa.10151](https://doi.org/10.1002/ajpa.10151)

Gould L, Sauther M. *Lemurs: Ecology and Adaptation*. 2006. Springer Press, New York.

Harcourt C. Diet and behaviour of a nocturnal lemur, Avahi laniger, in the wild. J Zool. 1991: 223: 667-674.

Irwin MT. Diademed sifaka (Propithecus diadema) ranging and habitat use in continuous and fragmented forest: Higher density but lower viability in fragments? Biotropica. 2007. Doi: 10.1111/j.1744- 7429.2007.00368.x.

Irwin MT, Glander KE,, Raharison JL, Samonds KE.. Effect of habitat and sex on body mass and morphometrics of diademed sifakas (Propithecus diadema). Am J Phys Anthropol. 2007; 132, (S44):134.

Johnson SE. *Ecology and speciation in brown lemurs: White-collared lemurs (Eulemur albocollaris) and hybrids (Eulemur albocollaris× Eulemur fulvus rufus) in southeastern Madagascar*. PhD Thesis, The University of Texas at Austin. 2002.

Johnson SE, Gordon AD, Stumpf RM, Overdorff DJ, Wright PC. Morphological variation in populations of *Eulemur albocollaris* and *E. fulvus rufus*. *Int J Primat*. 2005: *26*: 1399-1416.

Kappeler PM, Fichtel C. The evolution of Eulemur social organization. Int J Primatol. 2016; *37*: 10-28.

Kobbe S, Ganzhorn JU, Dausmann KH. Extreme individual flexibility of heterothermy in free-ranging Malagasy mouse lemurs (Microcebus griseorufus). *J Comp Physiol B*. 2011; *181*: 165-173.

Koch F, Ganzhorn JU, Rothman JM, Chapman CA, Fichtel C. Sex and Seasonal differences in diet and nutrient intake in Verraux’s sifaka (Propithecus verreaxi). Am J Primatol. 2017; 79:1-10. [**doi: 10.1002/ajp.22595**](https://doi.org/10.1002/ajp.22595)

Martinez BT, Razafindratsima OH. Frugivory and Seed Dispersal Patterns of the Red-Ruffed Lemur, Varecia rubra, at a Forest Restoration Site in Masoala National Park, Madagascar. Folia Primatol. 2014; 85: 228–243.

Mittermeier RA, Valladares-Pádua C, Rylands AB, Eudey AA, Butynski TM, Ganzhorn JU, Kormos R, Aguiar JM, Walker S. Primates in peril: the world's 25 most endangered primates, 2004–2006. Prim Conserv. 2006; 20:1-28.

Lahann P. Feeding ecology and seed dispersal of sympatric cheirogaleid lemurs (Microcebus murinus, Cheirogaleus medius, Cheirogaleus major) in the littoral rainforest of south-east Madagascar. J Zool. 2007; 271: 88-98. Doi: 10.1111/j.1469-7998.2006.00222.x

Lahann P, Dausmann, KH. Live Fast, Die Young: Flexibility of Life History Traits in the Fat-Tailed Dwarf Lemur (Cheirogaleus Medius). Beh Ecol Sociobiol. 2011; 65: 381-190.

Lahann P, Schmid J, Ganzhorn JU. Geographic variation in populations of Microcebus murinus in Madagascar: resource seasonality or Bergmann's rule?. *Int J Primatol. 2006;* 27: 983-999.

Lewis RJ, Kappelar PM. Seasonality, Body Condition, and Timing of Reproduction in Propithecus verreauxi verreauxi in the Kirindy. Am J Primatol. 2005; 67: 347-364.

Norscia I, Ramanamanjato JB, Ganzhorn JRU. Feeding Patterns and Dietary Profile of Nocturnal Southern Woolly Lemurs (Avahi meridionalis) in Southeast Madagascar. Int J Primatol. 2011; 33: 150–167. [doi](http://en.wikipedia.org/wiki/Doi_(identifier)" \o "Doi (identifier)): [10.1007/s10764-011-9562-3](https://doi.org/10.1007%2Fs10764-011-9562-3). [S2CID](http://en.wikipedia.org/wiki/S2CID_(identifier)) [14395500](https://api.semanticscholar.org/CorpusID:14395500).

Norscia I. Pilot survey of avahi population (woolly lemurs) in littoral forest fragments of southeast Madagascar. Primates. 2008; 49: 85–88. [doi](http://en.wikipedia.org/wiki/Doi_(identifier)):[10.1007/s10329-007-0061-2](https://doi.org/10.1007%2Fs10329-007-0061-2).

Overdorff DJ. Similarities, differences, and seasonal patterns in the diets of Eulemur rubriventer and Eulemur fulvus rufus in the Ranomafana National Park, Madagascar. Int J Primatol. 1993; 14*:* 721–753*.*

Overdorff DJ, Strait SG, Telo A. Seasonal variation in activity and diet in a small-bodied folivorous primate, Hapalemur griseus, in southeastern Madagascar. *Am J Primatol*. 1997; 43: 211-223.

Pereira ME, Seeligson ML, Macedonia JM. The behavioral repertoire of the black-and-white ruffed lemur, Varecia variegata variegata (Primates: Lemuridae). Folia Primatol. *1988; 51: 1*-32.

Perret M, Aujard EF. Regulation by photoperiod of seasonal changes in body mass and reproductive function in gray mouse lemurs (Microcebus murinus): differential responses by sex. *Int J Primatol. 2001; 22*: 5-24.

Pride RE. Optimal group size and seasonal stress in ring-tailed lemurs (Lemur catta), Behav Ecol. 2005; 16: 550–560. Doi: 1[0.1093/beheco/ari025](https://doi.org/10.1093/beheco/ari025)

Radespiel U. Sociality in the gray mouse lemur (Microcebus murinus) in northwestern Madagascar. *Am J Primatol. 2000;* *51*: 21-40.

Rakotondrina AJV, Andriantsimanarilafy RR, Andrianarivelo JF, Benjamin LA, Zaonarivelo JR, Ratsimbazafy J. Population Assessment of the Crowned Lemur (Eulemur coronatus) in the Bobaomby Area, Northern Madagascar. Prim Conserv. 2023; 37: DOI

Rahlfs M, Fichtel C. Anti-predator behaviour in a nocturnal primate, the grey mouse lemur (Microcebus murinus). Ethology. 2010; 116: 429-439. Doi: 10.1111/j.1439-0310-2010.01756.x

##### Ralainasolo FB, Ratsimbazafy JH, Stevens N J. Behavior and diet of the Critically Endangered Eulemur cinereiceps in Manombo forest, southeast Madagascar. *Madagascar Conservation & Development*. 2008; *3*: doi: 10.4314/mcd.v3i1.44134

Ralison JM. The lemurs of the Ambatovy-Analamay region. *Biodiversity, exploration, and conservation of the natural habitats associated with the Ambatovy project. Malagasy Nature*. 2010; *3*: 178-191.

Randimbiharinirina DR, Raharivololona BM, Hawkins MT, Frasier CL, Culligan RR, Sefczek TM, Randriamampionona R, Louis JrEE. Behaviour and Ecology of Male Aye-Ayes (Daubentonia madagascariensis) in the Kianjavato Classified Forest, South-Eastern Madagascar. Folia Primatol. 2018; 89: 123-137. [doi: 10.1159/000486673](https://doi.org/10.1159/000486673)

Rasolooarison RM, Goodman GM, Ganzhorn JU. Taxonomic revision of mouse lemurs (Microcebus) in the western portions of Madagascar. *Int J Primatol. 2000;*, *21*: 963-1019.

Richard AF. Social boundaries in Malagasy prosimian, the sifaka (Propithecus verreauxi). Int J Primatol. 1985; 6: 553-568. Doi: 10.1007/BF02692288.

Rigamonti MM. Home range and diet in red ruffed lemurs (Varecia variegata rubra) on the Masoala Peninsula, Madagascar. In: Kappelar PM,

Ganzhorn JU, editors. Lemur social systems and their ecological basis. Springer, Boston, MA; 1993. Doi: 10.1007/978-1-4899-2412-4_3.

Rodriguez IA, Rasoazanabary E, Godfrey LR. Seasonal variation in the abundance and distribution of ticks that parasitize Microcebus griseorufus at the Bezà Mahafaly Special Reserve, Madagascar. Int J Parasit: Parasites and Wildlife. 2015; 4: 408-413. Doi: 10.1016/j.ijppaw.2015.10.007.

Roen I. Things that go 'Munch' in the Night: Behavior, Range, and Feeding Ecology of a Mother and Offspring Daubentonia Madagascariensis. ISP thesis. 2014. Available at: <https://digitalcollections.sit.edu/isp_collection/1764>

Royo J, Aujard F, Pifferi F. Daily torpor and sleep in a non-human primate, the gray mouse lemur (Microcebus murinus). *Frontiers in Neuroanatomy. 2019;* *13*: 87.

Sato H. Habitat shifting by the common brown lemur (*Eulemur fulvus fulvus*): a response to food scarcity. *Primates.* 2013; 54**:** 229–235. https://doi.org/10.1007/s10329-013-0353-7

Sato, Ichino S, Hanya G. Dietary modification by common brown lemurs (Eulemur fulvus) during seasonal drought conditions in western Madagascar. *Primates*. 2014; *55*: 219-230.

Schmidt M. Locomotion and postural behaviour, Adv. Sci. Res. 2010; 5: 23–39. Doi: 1[0.5194/asr-5-23-2010](https://doi.org/10.5194/asr-5-23-2010)

Schmid J, Ruf T, Heldmaier G. Metabolism and temperature regulation during daily torpor in the smallest primate, the pygmy mouse lemur (*Microcebus myoxinus*) in Madagascar. J Comp Physiol B 2000; 170:59–68

Schmid J, Kappeler PM. Fluctuating sexual dimorphism and differential hibernation by sex in a primate, the gray mouse lemur (Microcebus murinus). *Beh Ecol Sociobiol. 1998; 43:* 125-132.

Schmid J. Daily torpor in the gray mouse lemur (Microcebus murinus) in Madagascar: energetic consequences and biological significance. *Oecologia*. 2000; *123*: 175-183.

Schülke O, Ostner J. Physiological ecology of cheirogaleid primates: variation in hibernation and torpor. *Acta Ethol. 2007; 10*: 13-21.

Schwab D. A preliminary study of spatial distribution and mating system of pygmy mouse lemurs (Microcebus cf myoxinus). *Am J Primatol. 2000;* *51*: 41-60.

Schwab D, Ganzhorn JU. Distribution, population structure and habitat use of Microcebus berthae compared to those of other sympatric cheirogalids. *Int J Primatol. 2004;* *25*: 307-330.

Sefczek TM, Hagenson RA, Randimbiharinirina DR, Rakotondrazandry JN, Louis Jr, EE. Home Range Size and Seasonal Variation in Habitat Use of Aye-Ayes (Daubentonia madagascariensis) in Torotorofotsy, Madagascar. Folia Primatol. 2020; 91: 558-574. [doi: 10.1159/000508620](https://doi.org/10.1159/000508620)

Simmen B, Bayart F, Marez A, Hladik A. Diet, nutritional ecology, and birth season of Eulemur macaco in an anthropogenic forest in Madagascar. Int J Primatol. 2007; 28: 1253-1266.

Sterling EJ. Patterns of Range Use and Social Organization in Aye-Ayes (*Daubentonia Madagascariensis*) on Nosy Mangabe. In: Kappeler PM, Ganzhorn, JU (eds) Lemur Social Systems and Their Ecological Basis. Springer, Boston, MA. 1993. Doi: [10.1007/978-1-4899-2412-4_1](https://doi.org/10.1007/978-1-4899-2412-4_1)

Sterling EJ, McCreless EE. Adaptations in the Aye-aye: A Review. In: Gould L, Sauther ML. (eds) Lemurs. Developments in Primatology: Progress and Prospect. Springer, Boston, MA. 2006. Doi: 10.1007/978-0-387-34586-4_8

Tan CL. Group Composition, Home Range Size, and Diet of Three Sympatric Bamboo Lemur Species (Genus *Hapalemur*) in Ranomafana National Park, Madagascar. *Int J Primatol.* 1999*;* 20: 547–566. https://doi.org/10.1023/A:1020390723639

Terranova CJ, Coffman BS. (1997). Body weights of wild and captive lemurs. *Zoo Biol. 1997;* *16*: 17-30.

Thalmann U. Food Resource Characteristics in Two Nocturnal Lemurs with Different Social Behavior: Avahi occidentalis and Lepilemur edwardsi. *Int J Primatol*. 2001; 22: 287–324. Doi: 10.1023/A:1005627732561

Thalmann U, Geissmann T. Distribution and Geographic Variation in the Western Woolly Lemur (Avahi occidentalis) with Description of a New Species (*A. unicolor*). *Int J Primatol*: 2000*;* 21, 915–941. Doi: 10.1023/A:1005507028567Warren and Crompton 1997

Thorén S, Quietzsch F, Schwochow D, Sehen L, Meusel C, Meares K, Radespiel U. Seasonal changes in feeding ecology and activity patterns of two sympatric mouse lemur species, the gray mouse lemur (Microcebus murinus) and the golden-brown mouse lemur (M. ravelobensis), in northwestern Madagascar. *Int J Primatol. 2011;* *32*: 566-586.

Vasey N. The breeding system of wild red ruffed lemurs (*Varecia rubra*): a preliminary report. *Primates. 2007;* 48: 41–54. [doi: 10.1007/s10329-006-0010-5](https://doi.org/10.1007/s10329-006-0010-5)Vasey N. Community ecology and behavior of Varecia variegata rubra and Lemur fulvus albifrons on the Masoala Peninsula, Madagascar. PhD Thesis. Washington University in St. Louis. 1997. Available from: <https://openscholarship.wustl.edu/etd_restrict/65/>

Volampeno SMN, Masters JC, Downs CT. Home range size in the blue-eyed black lemur (Eulemur flavifrons): A comparison between dry and wet seasons. *Mammal Biol. 2011;*, *76*:157-164.

Volampeno MSN, Downs CT, Randriatahina G. Structure and composition of Ankarafa Forest, Sahamalaza-Iles Radama National Park, Madagascar: Implications for the frugivorous endemic blue-eyed black lemur (Eulemur flavifrons). *South African J Wildlife Research. 2013; 43*: 91-102.

Warren RD, Crompton RH. A comparative study of the ranging behaviour, activity rhythms and sociality of *Lepilemur edwardsi* (Primates, Lepilemuridae) and *Avahi occidentalis* (Primates, Indriidae) at Ampijoroa, Madagascar. *J. Zool*. 1997; 243: 397–415.

White FJ, Overdorff DJ., Balko EA,.Wright PC. Distribution of ruffed lemurs (Varecia variegata) in Ranomafana National Park, Madagascar. *Folia Primatol. 1995*; *64*: 124-131.

Wilson JM, Stewart PD, Ramangason GS, Denning AM, Hutchings MS. Ecology and conservation of the crowned lemur, Lemur coronatus, at Ankarana, N. Madagascar. *Folia Primatol. 1989*; *52*: 1-26.

Wright PC, Martin LB. Predation, pollination and torpor in two nocturnal primates: Cheirogaleusmajor and Microcebus rufus in the rain forest of Madagascar. In: Alterman L, Doyle G, Izard K. editors. Creatures of the Dark: The Nocturnal Prosimians. New York: Plenum Press. 2005. pp. 45-60.

Wright, P. C, & Martin, L. B. (1995). Predation, pollination and torpor in two nocturnal primates: Cheirogaleus

major and Microcebus rufus in the rain forest of Madagascar. In L. Alterman, G. Doyle, & K. Izard

(eds.), Creatures of the Dark: The Nocturnal Prosimians (pp. 45–60). New York: Plenum Pres

Zaramody A, Fausser JL, Roos C, Zinne D, Andriaholinirina N, Rabarivola C, Norsci I, Tattersall I, Rumpler Y. Molecular phylogeny and taxonomic revision of the eastern woolly lemur (*Avahi laniger*). Prim Report. 2006; 74: 9-22.
